# A hybrid system for the overproduction of complex ergot alkaloid chanoclavine

**DOI:** 10.3389/fbioe.2022.1095464

**Published:** 2022-12-21

**Authors:** Yaqing Ma, Juzhang Yan, Lujia Yang, Yongpeng Yao, Luoyi Wang, Shu-Shan Gao, Chengsen Cui

**Affiliations:** ^1^ CAS Key Laboratory of Microbial Physiological and Metabolic Engineering, State Key Laboratory of Microbial Resources, Institute of Microbiology, Chinese Academy of Sciences, Beijing, China; ^2^ Tianjin Institute of Industrial Biotechnology, Chinese Academy of Sciences, Tianjin, China; ^3^ University of Chinese Academy of Sciences, Beijing, China; ^4^ National Technology Innovation Center of Synthetic Biology, Tianjin, China

**Keywords:** ergot alkaloid, cell-lysate catalysis, chemical synthesis, whole-cell catalysis, Sbio-Csyn system

## Abstract

Synthetic biology-based methods (Sbio) and chemical synthesis (Csyn) are two independent approaches that are both widely used for synthesizing biomolecules. In the current study, two systems were combined for the overproduction of chanoclavine (CC), a structurally complex ergot alkaloid. The whole synthetic pathway for CC was split into three sections: enzymatic synthesis of 4-Br-Trp (4-Bromo-trptophan) using cell-lysate catalysis (CLC), chemical synthesis of prechanoclavine (PCC) from 4-Br-Trp, and overproduction CC from PCC using a whole-cell catalysis (WCC) platform. The final titer of the CC is over 3 g/L in this Sbio-Csyn hybrid system, the highest yield reported so far, to the best of our knowledge. The development of such a combined route could potentially avoid the limitations of both Sbio and Csyn systems and boost the overproduction of complex natural products.

## 1 Introduction

**FIGURE 1:**
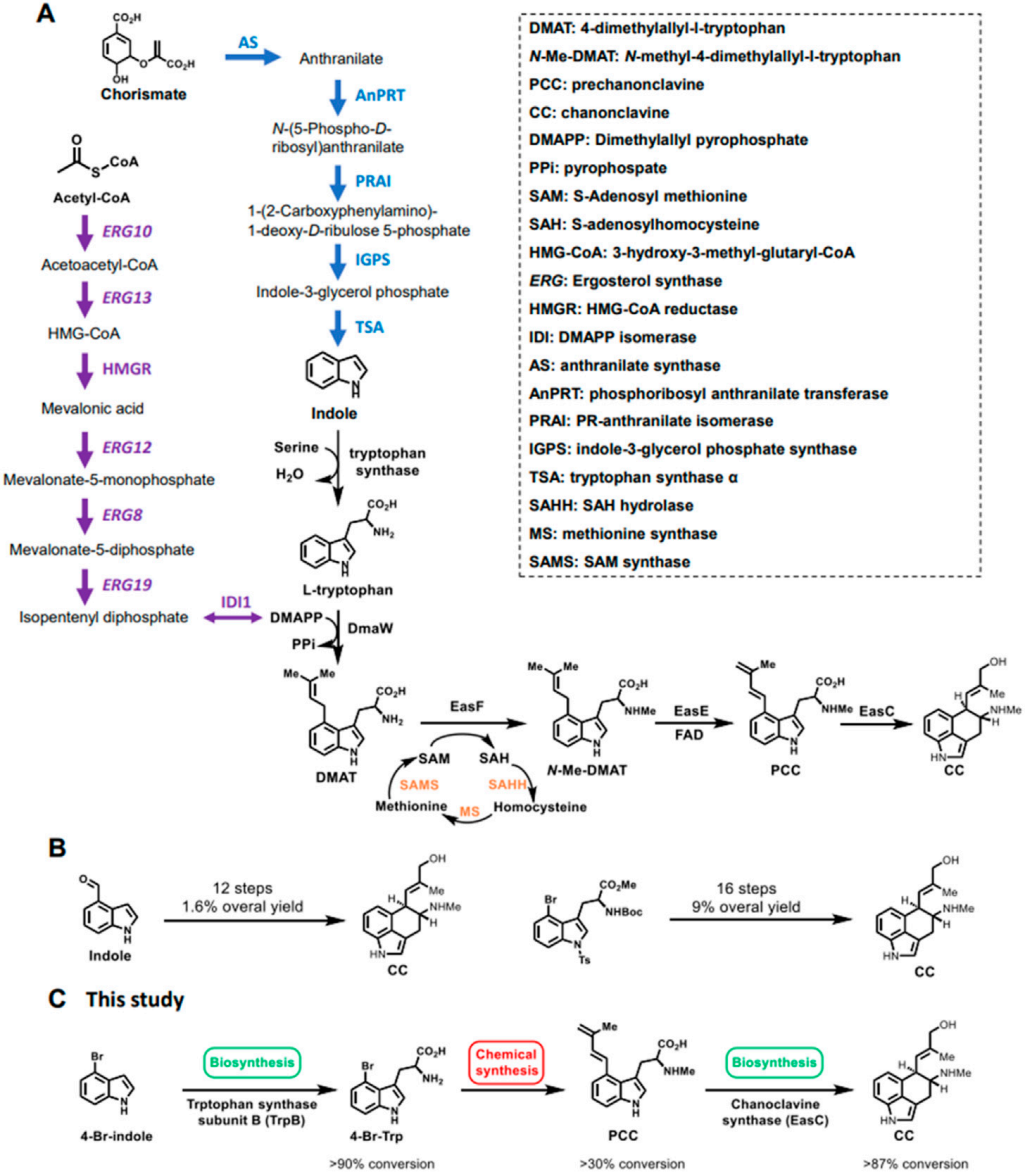
The biosynthetic pathway of chanoclavine (CC). **(A)** The biosynthetic pathway of CC in the native hosts. **(B)** Chemical synthesis of CC in the literature. **(C)** The hybrid route designed for the overproduction of CC in the current study.

Ergot alkaloids (EAs) are a group of highly bioactive natural products produced by a large number of filamentous fungi (Jakubczyk et al., 2014). These compounds have been intensely studied for decades, mainly due to their harmful effects on contaminated food and feeds, but also for their beneficial medicinal applications. For instants, EA compound chanoclavine (CC, Figure 1A) can stimulate dopamine receptor D2 in the mouse brain (Watanabe et al., 1987) and has been long-term used in herbal drugs (Ysrael, 2003). Furthermore, CC is the common biosynthetic intermediate for the biosynthesis of almost all EAs (Jakubczyk et al., 2015a).

The biosynthesis of CC requires three precursors: tryptophan, dimethylallyl pyrophosphate (DMAPP), and S-adenosyl methionine (SAM) (Gerhards et al., 2014; Jakubczyk et al., 2014). In the fungal cells, the shikimic acid pathway responsible for the tryptophan biosynthesis contains six enzymes (Figure 1A), whereas the mevalonate pathway for DMAPP production has seven enzymes (Figure 1A) (Zhao et al., 2013). Furthermore, the regeneration of SAM requires three enzymes (Figure 1A) (Parveen & Cornell, 2011). Starting from the three precursors, three prenylated-tryptophan intermediates, dimethylallyltryptophan (DMAT), *N*-methyldimethylallyltryptophan (*N*-Me-DMAT), and prechanoclavine (PCC), are generated under the successive catalysis of prenyltransferase DmaW, methyltransferase EasF, and FAD-linked oxidoreductase EasE (Gerhards et al., 2014; Jakubczyk et al., 2014). The final enzyme for converting PCC to CC remained unknown until recently when we characterized chanocalvine synthase EasC responsible for this step of oxidative cyclization (Nielsen et al., 2014; Yao et al., 2019). Overall, 20 enzymes are involved in the biosynthesis of CC in the native host (Figure 1A).

Accompanied by the complexity of the biosynthetic pathways, the construction and engineering of synthetic biology (Sbio) based methods for overproducing natural products, including microbial cell factories or cell-free systems, become increasingly difficult. As the biosynthesis of CC employed many enzymes for precursor production and tailoring modifications (Figure 1A), it led to difficulties in overproducing CC using microbial cell factories or cell-free systems. The previous production of CC in *S. cerevisiae* and *Aspergillus nidulans* reached the titers at 1.2 and 241.0 mg/L, respectively (Nielsen et al., 2014; Yao et al., 2022), which was still far from industrial application. Furthermore, our recent studies demonstrated the effort of purification of soluble EasE from *Escherichia coli*, *S. cerevisiae,* or *Trichoderma reesei* was not successful, suggesting it was not likely to use cell-free system to overproduce CC from tryptophan. In conclusion, upgrading the current Sbio system is necessary for improving the titers of CC.

On the other hand, the chemical synthesis (Csyn) method for CC production has also been developed. CC possess complex conjugated rings with several stereocenters (Figure 1B), which led to their total synthesis being lengthy, inefficient, and not profitable (Liu & Jia, 2017). Previous research has reported two strategies for asymmetric total syntheses of CC (Kardos & Genet, 1994; Yokoyama et al., 1996), which started from complex 4-substituted indole derivatives using a 12-step and 16-step process, respectively. In addition to the shortage of expensive starting materials, these routes were inefficient for industrial applications, with 1.6% and 9% overall yield, respectively, and more research was needed to increase their efficiency.

To avoid the limitations that arise from both Csyn- and Sbio-based systems, and to take full advantage of both systems, we developed a combined system to synthesize clinically significant and structurally complex CC in this study (Figure 1C). The combined system that hired both Csyn- and Sbio-based methods could efficiently synthesize CC, with a titer of over 3 g/L and an engineering period of up to 1 week. Thus, the combined Sbio-Csyn system represents a fast, robust, and practical engineering methodology for laboratory and industrial applications.

## 2 Materials and methods

### 2.1 General materials and methods

The authentic compounds prechanoclavine and agroclavine were purified and characterized, which were stored in our laboratory. All vectors and strains, including those vectors for EasC and TrpB expression, used in this study were stored in our laboratory. Electroporation was performed on A Bio-Red MicroPulser (Bio-Red 1652100, United States). The chemicals and solvents purchased and used in this study were analytical grade. All buffers and solutions were prepared with Milli-Q water. DNA sequencing and primer synthesis were done by Tsingke Biotechnologies (Beijing, China). Restriction enzymes and Q5 DNA polymerase were purchased from New England Biolabs (United States). HPLC grade acetonitrile and water were purchased from Sigma-Aldrich (St. Louis, MO, United States). A CORUI HPLC system (Chengdu, China) using C18 analytical column (Agilent Eclipse XDB-C18, 4.6 × 250 mm, 5 μm) was used to perform high performance liquid chromatography (HPLC) analysis.

### 2.2 Plasmid construction

The cDNAs of EasC from *Aspergillus japonicus* and Tm2F3 were synthesized for the heterologous protein expression, which were inserted into the site NdeI/XhoI of pET28a by the Gibson method to generate the corresponding plasmids. The resultant plasmids were transformed into the competent cells of *E. coli* BL21 (DE3) and plated on LB solid medium with 50 mg/ml kanamycin at 37°C overnight to get the correct transformants.

### 2.3 Chemical synthesis of PCC

^1^H NMR spectra were recorded in CDCl_3_, CD_3_OD, (CD_3_)_2_SO (400 or 600 MHz). Residual solvent peaks are used as the internal reference; the signals at 7.26 ppm are set for ^1^H NMR spectra, taken in CDCl_3_. Silica gel plates pre-coated on glass were used for thin-layer chromatography using UV light, or 7% ethanolic phosphomolybdic acid or potassium permanganate solution and heating as the visualizing methods. Silica gel was used for flash column chromatography with mixed CH_2_Cl_2_ and MeOH or ethyl acetate (EtOAc) and hexane as the eluting solvents. Yields refer to chromatographically and spectroscopically (^1^H NMR) homogeneous materials. All reactions were performed under an oxygen-free atmosphere of nitrogen or argon, unless otherwise stated. Reagents were obtained commercially and used as received unless otherwise mentioned. Anhydrous THF, Et_2_O and PhMe were freshly distilled from sodium and benzophenone ketyl and anhydrous DMA, DMF, CH_2_Cl_2_ and CH_3_CN were freshly distilled over CaH_2_, respectively, under a Ar_2_ atmosphere. Room temperature is 23 °C unless otherwise stated.

### 2.4 HPLC and LC-MS analysis

HPLC analysis was performed on CORUI HPLC system (Chengdu, China) using C18 columns (Agilent Eclipse XDB-C18, 4.6 × 250 mm, 5 μm) and a Photodiode Array Detector. The samples of a 10 μL injection volume were analyzed with a linear gradient method of 90%–10% H_2_O (v/v, 0.1% formic acid) -acetonitrile (v/v, 0.1% formic acid) to 100% acetonitrile (v/v, 0.1% formic acid) in 10 min with a flow rate of 1.0 ml/min.

LC-MS analysis was performed on Agilent 1,100 with a mass spectrum detector (MSD) using an analytical column (Ultimate XB-C18, 2.1 × 100 mm, 3.0 μm; Welch), and the positive ion mode was used to perform the mass spectrometry. The samples of a 1.0 μL injection volume were analyzed with a linear gradient method of 90%–10% H_2_O (v/v, 0.1% formic acid) -acetonitrile (v/v, 0.1% formic acid) to 100% acetonitrile (v/v, 0.1% formic acid) in 12 min with a flow rate of 0.3 ml/min.

### 2.5 Strain culture and biotransformation for the biosynthesis of CC

10 ml LB liquid medium containing 50 μg/ml kanamycin was inoculated with a correct transformant of EasC and cultured at 37°C and 220 rpm overnight for 12 h. The overnight culture was used a seed medium and transferred into 1L LB liquid medium and incubated at 37°C with shaking at 220 rpm to an OD_600_ of 0.6–0.8. Protein expression was induced by the addition of 10 μM isopropyl-β-d-thiogalactopyranoside (IPTG) and 5-aminolevulinic acid (5-ALA, 80 mg/L) and shaking was continued for 18 h at 16°C, 200 rpm. The bacteria were then harvested by centrifugation at 5,000 rpm for 20 min and resuspended in 50 ml sodium phosphate buffer (PBS, 50 mM, pH 7.4).

For the reaction with whole cells, different concentrations of substrate PCC and 2 eq NADPH were added to 500 μL of the above bacterial suspension, and then incubated at 30°C with shaking at 800 rpm. For the reaction with cell lysate, the bacterial suspension was firstly disrupted by ultrasonication (30 min with 5 s' on, 9 s' off cycles), then different concentrations of PCC and 2 eq NADPH were added to 500 μL of the disruption solution followed by incubating at 30°C, 800 rpm. The reactions were sampled at different times, quenched with methanol and checked for conversion with HPLC. In addition, three parallels were set for each concentration.

### 2.6 Enzymatic synthesis of 4-Br-Trp

Plasmid pET28a-Tm2F3 were transformed into *E. coli* BL21 (DE3) for expression the tryptophan synthase subunit B (Trp B). 8 × 10 ml LB medium containing 50 μg/ml kanamycin was inoculated with a single colony in a shaker at 37 °C overnight. The overnight cultures were used to inoculate 8×1L LB medium and shaken at 37 °C to an OD_600_ of 0.6–0.8. Then 0.5 mM IPTG was added and induced at 16 °C for 16 h. The cells were harvested by centrifugation (5,000 rpm at 4 °C for 20 min), and the cell pallets were resuspended in 100 ml lysis buffer (100 mM PBS, pH 8.0) and disrupted by high pressure cell fragmentation apparatus (Guangzhou Juneng Biotechnology Co., LTD). To remove cellular debris, the mixture was centrifuged at 12,000 rpm for 30 min at 4°C. The supernatant was transferred into a 250 ml Erlenmeyer flask, 4-Br-indole (dissolved in DMSO) and serine were added into the reaction system. The reaction mixture was immersed in a metal bath that was pre-heated to 75°C for 24 h and then cooled to 0°C (ice bath), in which most of 4-Br-Trp precipitated. Subsequently, the reaction mixture was centrifuged at 12,000 rpm for 20 min, and the precipitate was washed with water three times and concentrated *in vacuo* to obtain 4-Br-Trp. The supernatant was subjected to the hollow fiber ultrafiltration membrane column to remove the protein, and the aqueous phase was concentrated *in vacuo* to obtain 4-Br-Trp.

## 3 Results

### 3.1 Determining the splitting point of the hybrid system

To design a combined Sbio-Csyn system, we selected PCC as the splitting point (Figure 1C). We split the whole synthetic pathway into three sections (Figure 1C): 1) enzymatic synthesis 4-Br-Trp from the cheap material 4-Br-indole using the tryptophan synthase subunit B (TrpB); 2) efficient chemical synthesis of PCC from 4-Br-Trp; and 3) overproduction of CC from PCC using the chanoclavine synthase EasC (Figure 1C). The key point of this study is to design a hybrid platform that combines the advantages of both Sbio- and Csyn-based methods to produce high-valued natural products from cheap starting materials.

### 3.2 Biosynthesis of 4-Br-Trp from 4-Br-indole

**FIGURE 2:**
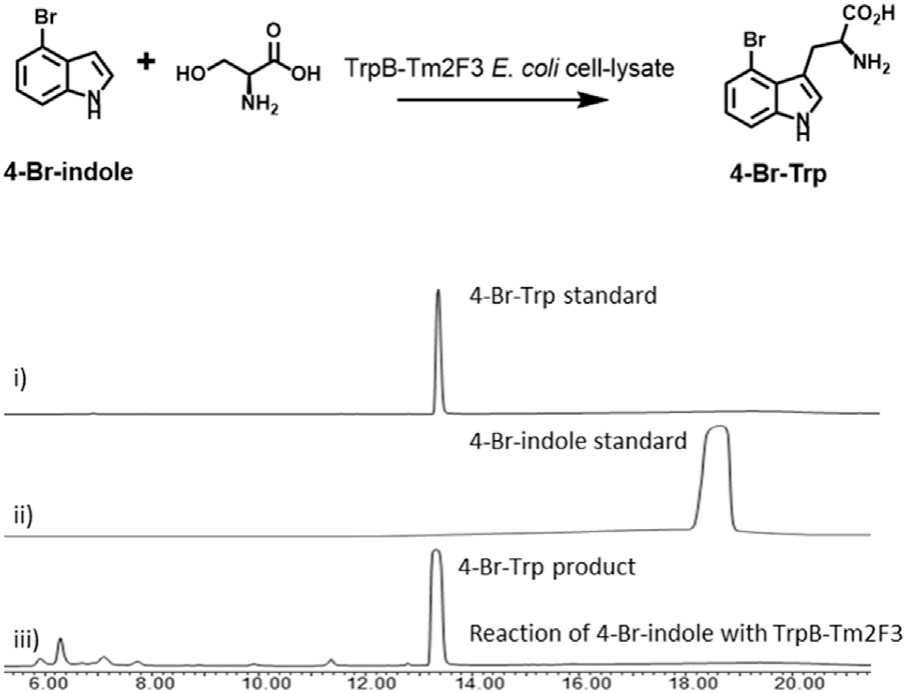
Gram-scale production of 4-Br-Trp using a cell-lysate catalysis (CLC) system: i) HPLC trace for 4-Br-Trp standard; ii) HPLC trace for 4-Br-indole standard; and iii) HPLC trace for the reaction of 4-Br-indole with TrpB-Tm2F3. Reaction conditions are 10 mM 4-Br-indole, 30 mM serine, 0.13 g/L PLP, and 100 ml cell-lysate of *E. coli* expressing TrpB-Tm2F3 extracted from 8L fermentation.

4-Substituted tryptophans, such as 4-Br-Trp, serve as precursors for the chemical and biological synthesis of complex structures with a wide range of medicinal applications. The enantioselective synthesis of 4-substituted tryptophan compounds is often complicated due to protection and deprotection steps. At the same time, enzymes have the potential to synthesize those products in fewer steps and with precise chemo- and stereoselectivity. A notable example is TrpB, a pyridoxal phosphate (PLP) dependent enzyme (Romney et al., 2017). TrpB is the subunit B of tryptophan synthase, which can be coupled with indole and l-serine to generate the corresponding tryptophan analogue with retention of enantiopurity (Figure 2) (Corr et al., 2016). Accordingly, the TrpB platform has the potential to provide direct access to a wide range of 4-substituted tryptophans from cheap indole analogues. Directed evolution toward TrpB isolated from *Thermotoga maritima* endows it with high activity toward non-native substrates, such as different substituted indoles. The resultant mutant Tm2F3 (P19G, I69V, K96L, P140L, N167D, L213P, T292S) is especially attractive because it shows high activity for synthesizing 4-Br-Trp directly from serine and the corresponding 4-Br-indole (Figure 2) (Romney et al., 2017). Accordingly, TrpB-Tm2F3 mutant was used to overproduce 4-Br-Trp in the current study.

In our study, the mutant Tm2F3 was expressed in *E. coli* with a relatively high level (20 mg–30 mg/L, Supplementary Figure S1). It could be purified by heat treatment at a temperature of up to 75 °C with enhanced solubility. The cell-lysate catalysis (CLC) was applied to overproduce 4-Br-Trp, by using the cell lysate collected from the 8-L scale *E. coli* cells. Thus, enantiopure 4-Br-Trp could be synthesized by feeding the starting materials 4-Br-indole (10 mM) and serine (30 mM), and 0.01 g/L PLP to the cell lysate of *E. coli* expressing TrpB-TmF3. The overall conversion rate of CLC was below 50%, which was significantly lower than the reported rate from the literature (Romney et al., 2017). Next, we wished to see if the conversion rate could be improved by supplementing a higher concentration of cofactor PLP. Therefore, the CLC system was tested with 0.13 g/L PLP, instead of the initially used 0.01 g/L. Gratifyingly, the overall conversion rate of 4-Br-indole was improved to over 90%. Finally, 4-Br-Trp was synthesized in gram-scale with the production of 2.1 g of 4-Br-Trp (75% isolated yield) from the CLC with 8-L *E. coli* cell lysate, which provided sufficient starting material for the following chemical synthesis.

### 3.3 Chemical synthesis of PCC from 4-Br-Trp

**FIGURE 3:**
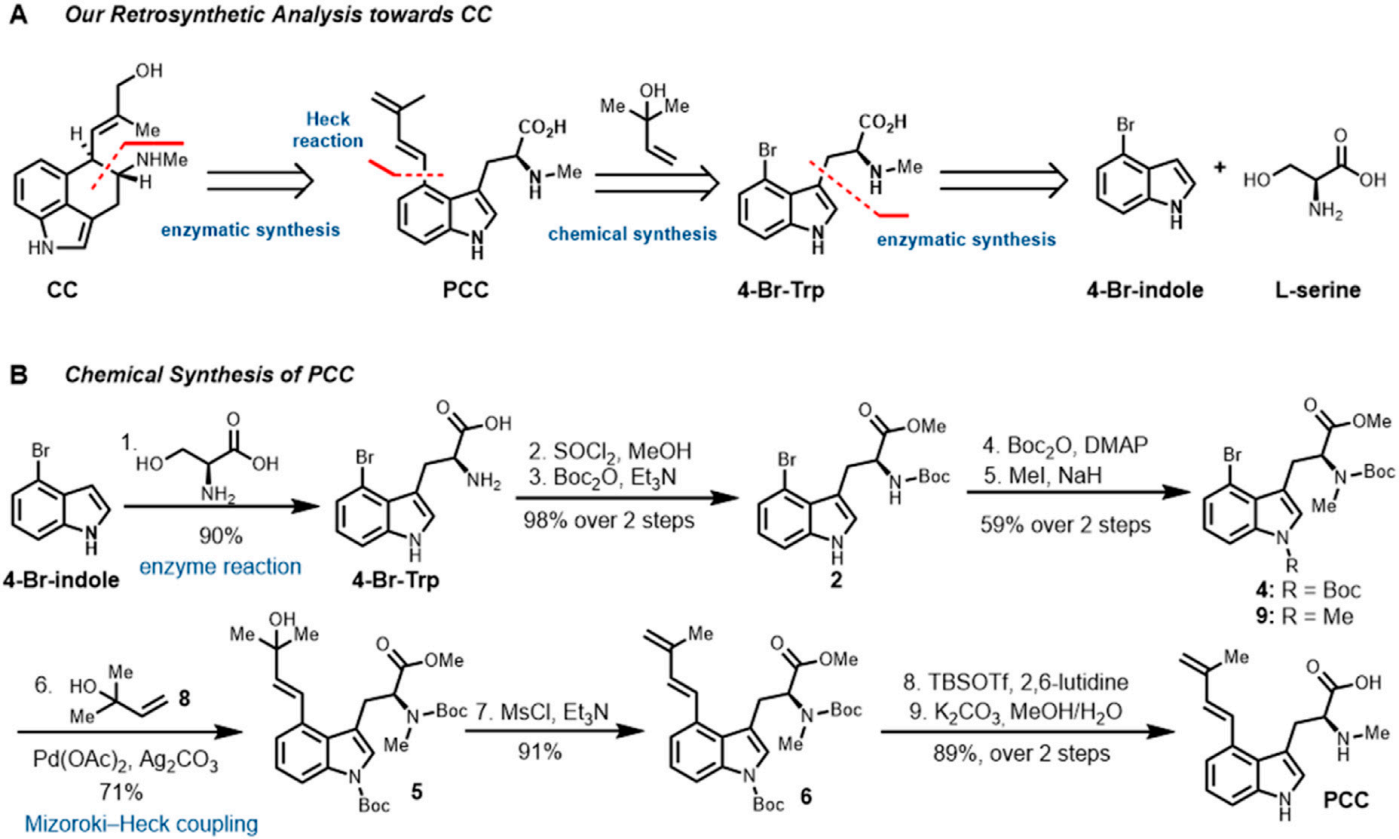
**(A)** Retrosynthetic bond disconnection. **(B)** Synthesis of PCC from 4-Br-Trp.

Retrosynthetically (Figure 3A), the C ring of CC could be constructed from compound PCC using EasC catalyzed reaction developed in our lab ((Yao et al., 2019); Figure 1A). The polyene side chain on PCC can be installed *via* Pd-catalyzed cross-coupling reactions such as Suzuki-Miyaura, Negishi, and Mizoroki-Heck coupling reactions (Figure 3A). To consider the commercial availability of starting material, 2-methyl-3-buten-2-ol **8** was chosen as an ideal fragment for Mizoroki-Heck coupling with 4-Br-Trp, which has already been synthesized in gram-scale *via* the CLC platform using the enzyme TrpB (Figure 2).

Our synthesis started with preparing Mizoroki-Heck coupling precursor 4-Br-Trp (Figure 3B), which was synthesized from commercially available 4-Br-indole. Enzyme catalyzed reaction with l-serine under our optimized conditions to give 4-Br-Trp in 90% yield. Esterification of 4-Br-Trp with SOCl_2_ in MeOH to obtain methyl ester in high yield. It should be noted that the esterification of 4-Br-Trp neither in Fisher condition (H_2_SO_4_, MeOH, reflux) nor base conditions (NaHCO_3_ and MeI in DMF, KHCO_3_ and MeI in MeOH, K_2_CO_3_ and MeI in MeCN, Cs_2_CO_3_ and MeI in MeOH) resulting decompose of the diene moiety. Initially, we tried to protect primary NH_2_ and indole nitrogen in one sequence, but unfortunately, a low yield of **3** (not shown in Figure 3B) was isolated. Then we turn to protect them separately, which was to protect free amine first by using Boc_2_O and Et_3_N conditions in Devaraj’s probe synthesis (Wu et al., 2014) to give a high yield of mono-protected product in 99% yield. Combining Boc_2_O with DMAP successfully protected the indole nitrogen in excellent yield, followed by methylation (NaH and MeI in DMF) (Ashworth et al., 1995) at secondary amine to afford compound **4** (59%, over two steps). In the methylation step, the Boc protecting group on indole nitrogen of product **4** could be selectively removed under basic condition, subsequent methylation with MeI to give side-product **9** in about 15% yield. The structure of side-product **9** was confirmed by ^1^H NMR and LCMS spectrum.

With **4** in hand, we then moved to explore the Mozoroki-Heck coupling with commercially available 2-methyl-3-buten-2-ol **8** outlined in Figure 3B. From the screening of conditions, Pd(OAc)_2_ and Ag_2_CO_3_ in toluene at 90 °C was identified as the reaction condition (Xu et al., 2010), which provided the highest conversion of **4** and yield of the desired coupling product **5** (71%). In parallel with the experimental exploration, we also tried the coupling conditions with AgOAc, K_2_CO_3_ in DMF or K_2_CO_3_ in DMF/H_2_O; it either provided the mass results or decomposed to the hydrolysis by-products. Notably, with PdCl_2_(PPh_3_)_2_ as a catalyst system, only a trace amount of desired **5** was detected.

Having established suitable conditions to achieve our target compound, tertiary alcohol **5** was converted into the diene moiety in a mesylation and elimination; the one-pot sequence gave **6** in excellent yield (91%) (Dethe et al., 2011). Deprotection of the N-Boc protecting group under the condition of TBSOTf, followed by ester hydrolysis (K_2_CO_3_ in MeOH/H_2_O), gave PCC in 89% yield over two steps. This synthesis can also be scaled up to multigram scale, thus providing reliable and safe access to PCC.

### 3.4 Overproduction of CC from PCC

With PCC in hand, we next aimed to synthesize CC by using EasC, a key enzyme that catalyzed the conversion of PCC to CC (Yao et al., 2019). To select the best ortholog of EasC, different EasC cDNAs were synthesized from *Aspergillus fumigatus*, *A. japonicus*, *Claviceps fusiformis*, *Periglandula ipomoeae*, *C. purpurea*, and *C. paspali* and introduced them into *E. coli* for protein purification (Yu et al., 2022), to evaluate their expression level. Our results indicated that EasC from *A. japonicus* (EasC_aj_) showed the highest protein expression level with ∼30 mg/L (Supplementary Figure S1). Accordingly, EasC_aj_ was applied in the current study for the overproduction of CC from PCC.

**FIGURE 4:**
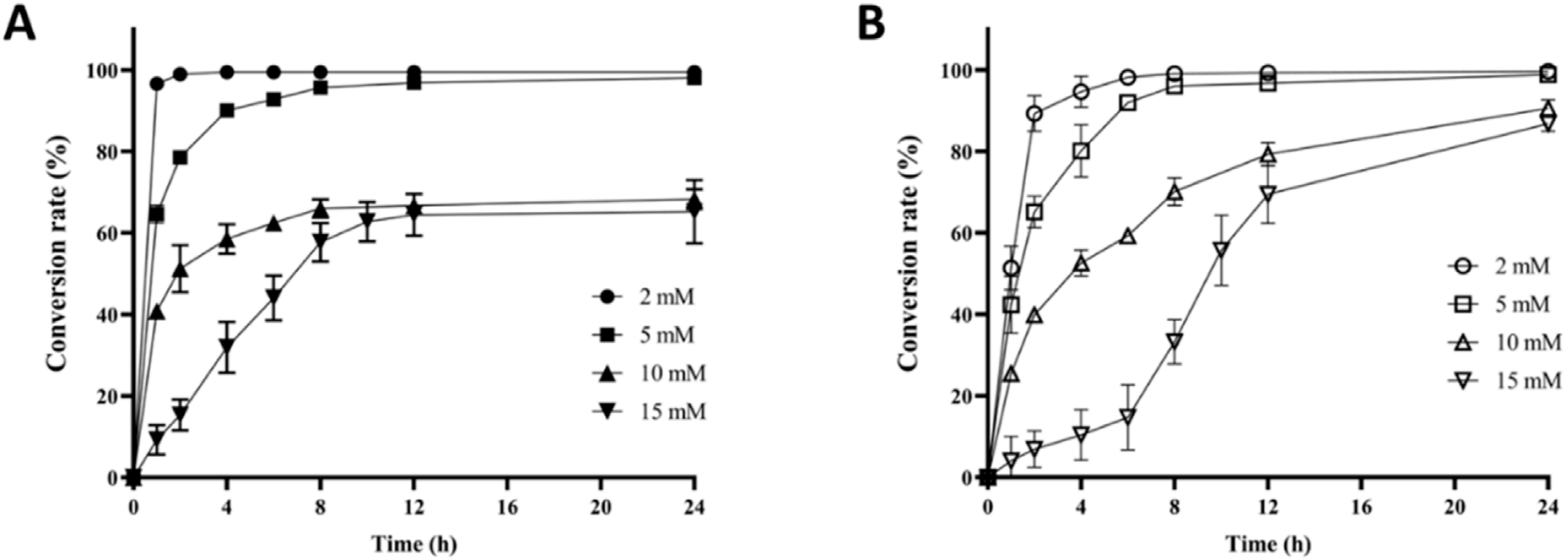
Overproduction of CC from PCC using two different methods. **(A)** The conversion rate of CC to PCC using EasC_aj_ based CLC platform. **(B)** The conversion rate of CC to PCC using EasC_aj_ based whole-cell catalysis (WCC) platform.

Firstly, the overproduction of CC was performed by applying CLC or whole-cell catalysis (WCC) using *E. coli* expressing EasC_aj_. According to the detected results (Figure 4), both CLC and WCC could completely transform 2 mM PCC to CC. However, the reaction rate of CLC was faster by converting >99% PCC to CC in 2 h, while 8 h was required for complete conversion through the latter method. Based on the above study, we attempted the biosynthesis of CC using higher concentrations of PCC. As shown in Figure 4, when the concentration of PCC was 5 mM, both methods completed the >95% conversion in 8 h.

However, when the concentration of PCC was doubled into 10 mM, the study showed different final conversion rates for the two methods. The catalytic rate of CLC was faster in the first 2 hours, which converted nearly 50% PCC to CC. However, compared to the 68% final conversion of CLC method, the WCC’s final conversion rate was significantly higher, with 91% conversion in 24 h’ reaction. The transformation after 2 h for the CLC method became extremely slow, suggesting that rapid protein deactivation had happened. Finally, 15 mM PCC was used for the biocatalytic synthesis of CC, and final conversions of 65% and 87% (∼3.34 g/L CC produced) could be obtained for CLC and WCC method, respectively. Similar to the reactions with 10 mM PCC, the CLC method showed a faster reaction rate in the first 8 h but with lower final conversion. In conclusion, based on the WCC platform using EasC_aj_ expressed in *E. coli*, we were able to overproduce CC with a titer of over 3 g/L.

## 4 Discussion

The combined Sbio-Csyn system established in this study seems superior to either the Csyn system or the Sbio system. The low levels of protein expression, high cost of cofactors and substrates, and multiple enzymes required make the overproduction of ergot alkaloids challenging to achieve *via* Sbio system (such as microbial cell factory or cell-free system). In accordance with this, no studies about cell-free systems have been reported for CC overproduction (Figure 4). Nielsen et al. first reported the microbial synthesis of CC in *Saccharomyces cerevisiae* with a titer of ∼1.2 mg/L (Nielsen et al., 2014). Later study indicated that this yeast strain with CC biosynthetic genes showed a higher final titer at low temperatures, likely due to the improved activity of the enzymes EasE and EasC (Jakubczyk et al., 2015b). Wong et al. also used yeast cells to produce CC heterologously, albeit with only detectable yield (Yan et al., 2022). The fungal platform *A. nidulans* was also applied for the biosynthesis of CC in several studies (Ryan et al., 2013; Yao et al., 2022). Ryan et al. described the production of CC by reconstituting its biosynthetic enzymes in *A. nidulans* in 2013 (Ryan et al., 2013). However, no final titer was reported. In 2022, Yao et al. used a Fungal-Yeast-Shuttle-Vector protocol to systematically refactor and engineer the CC biosynthetic pathway in *A. nidulans*, which led to a final titer of CC up to 241 mg/L (Figure 1A). In conclusion, the current protocols for the microbial synthesis of CC are not profitable for industrial production. To overcome those dilemmas, a combined Sbio-Csyn system was developed for the overproduction of CC. In the current study, the use of engineered TrpB smartly overproduced the expensive 4-Br-Trp from the cheap material 4-Br-indole in a Gram scale; through the chemical synthesis, the total synthesis of PCC from 4-Br-Trp was efficient and scalable by achieving >30% yield, and future optimization could further improve its overall yield. The final WCC platform with chanocalvine synthase EasC from *A. japonicus* led to the highest tier of CC (over 3 g/L, Figure 4) in the literature to the best of our knowledge.

Due to the abundance of minor metabolites, the isolated ergot alkaloids from ergot fermentation could be impure, leading to the industrial production of half amount of ergot alkaloid based on a field-production mode (Hanosova et al., 2015). Such difficulty could be exacerbated for those alkaloids with a low natural abundance, such as CC, typically transient intermediates in native EA-producers. Thus, developing a new mode to synthesize EAs could benefit the production of ergot-based pharmaceuticals. In this study, we developed a Sbio-Csyn hybrid system to overproduce the tricyclic CC with a titer of over 3 g/L. Our engineered systems overcame several major limitations that the current protocols for producing CC must be suffered, like strain degenerations and long growth cycles. For example, the present study of the field production for CC required months for the rye growth and harvest (Hulvova et al., 2013). In addition, *Claviceps* strains typically retained CC in its sclerotia (Hulvova et al., 2013). Instead, the *E. coli* strain for the WCC platform could secrete CC to the growth medium in our work, which would accelerate the isolation and purification of CC. Furthermore, *Claviceps* strains used for submerged fermentations suffered a degeneration process, which could reduce CC production frequently (Hulvova et al., 2013). Thus, our work will inject new power into the industrial production and medicinal application of EA-based drugs in the future. Future optimization of this combined system would improve the CC production and accelerate the industrial production of this medicinally crucial natural product. The key step in designing such a combined Sbio-Csyn system is to select a suitable splitting point between the two methods. We believe that the concept of the hybrid system could be further applied to the overproduction of more structurally complex natural products and pharmaceutical molecules, which are currently inaccessible through either the Sbio-or Csyn-based protocols.

## Data Availability

The original contributions presented in the study are included in the article/Supplementary Material, further inquiries can be directed to the corresponding authors.
