# Supplementary material for: A hybrid system for the overproduction of complex ergot alkaloid chanoclavine
Source: Front Bioeng Biotechnol. 2022 Dec 21;10:1095464. doi: 10.3389/fbioe.2022.1095464 (PMC9811125; doi:10.3389/fbioe.2022.1095464)
Supplement: Supplementary file 1 [file DataSheet1.pdf]

A Hybrid System for the Overproduction of Complex Ergot Alkaloid Chanoclavine

Yaqing Ma[a,b,c], Juzhang Yan[b], Lujia Yang[b], Yongpeng Yao[a], Luoyi Wang[a]*, Shu-Shan Gao[b,d]*, Chengsen Cui[b,d]*

[a]CAS Key Laboratory of Microbial Physiological and Metabolic Engineering, State Key Laboratory of Microbial Resources, Institute of Microbiology, Chinese Academy of Sciences, Beijing, China

[b]Tianjin Institute of Industrial Biotechnology, Chinese Academy of Sciences, Tianjin 300308, China

[c]University of Chinese Academy of Sciences, Beijing 100049, P.R. China

[d]National Technology Innovation Center of Synthetic Biology, Tianjin 300308, China

Table of Contents

Experimental Procedures. General Experimental……………………………………………S2
Table 1. Strains and plasmids used in the study…………………………………………….S3
Scheme 1. Chemical synthesis of PCC from 4-Br-Trp………………………………………….S4
References.……………………………………………………………………………………S9
^{1}H NMR spectra………………………………………………………………………………S10
Figure S1. The SDS-PAGE result of purified Tm2F3 and $EasC_{aj}$……………………………...S15

General Experimental

^{1}H NMR spectra were recorded in $CDCl_3$, CD_3OD, $(CD_3)_2SO$ (400 or 600 MHz). Residual solvent peaks are used as the internal reference; the signals at 7.26 ppm are set for ^{1}H NMR spectra, taken in $CDCl_3$. Silica gel plates pre-coated on glass were used for thin-layer chromatography using UV light, or 7% ethanolic phosphomolybdic acid or potassium permanganate solution and heating as the visualizing methods. Silica gel was used for flash column chromatography with mixed CH_2Cl_2 and MeOH or ethyl acetate (EtOAc) and hexane as the eluting solvents. Yields refer to chromatographically and spectroscopically (^{1}H NMR) homogeneous materials. All reactions were performed under an oxygen-free atmosphere of nitrogen or argon, unless otherwise stated. Reagents were obtained commercially and used as received unless otherwise mentioned. Anhydrous THF, Et_2O and PhMe were freshly distilled from sodium and benzophenone ketyl and anhydrous DMA, DMF, CH_2Cl_2 and CH_3CN were freshly distilled over CaH_2, respectively, under a Ar_2 atmosphere. Room temperature is 23 °C unless otherwise stated.

Table 1. Strains and plasmids used in the study

**Strains/plasmids**	**Description**	**Sourse**
Escherichia coli BL21(DE3)	F^- ompT hsdS(r_B^- m_B^-) gal dcm(DE3)	Zoman Biotechnology Co., Ltd. (Beijing, China)
pET-28a-Tm2F3	The mutant Tm2F3 (P19G, I69V, K96L, P140L, N167D, L213P, T292S) of TrpB from *Thermotoga maritima* cloned in pET-28a+ by 5' Ndel- 3' Xhol	
pET-28a-EasC$_{af}$	The gene of wild-type EasC from *Aspergillus fumigatus* cloned in pET-28a+ by 5' Ndel- 3' Xhol	
pET-28a-EasC$_{aj}$	The gene of wild-type EasC from *A. japonicus* cloned in pET-28a+ by 5' Ndel- 3' Xhol	
pET-28a-EasC$_{c.fus}$	The gene of wild-type EasC from *Claviceps fusiformis* cloned in pET-28a+ by 5' Ndel- 3' Xhol	Tsingke Biotechnology Co., Ltd. (Beijing, China)
pET-28a-EasC$_{pi}$	The gene of wild-type EasC from *Periglandula ipomoeae* cloned in pET-28a+ by 5' Ndel- 3' Xhol	
pET-28a-EasC$_{c.pur}$	The gene of wild-type EasC from *C. purpurea* cloned in pET-28a+ by 5' Ndel- 3' Xhol	
pET-28a-EasC$_{c.pas}$	The gene of wild-type EasC from *C. paspali* cloned in pET-28a+ by 5' Ndel- 3' Xhol	

The detailed route and conditions

Scheme S1. Chemical synthesis of PCC from 4-Br-Trp.

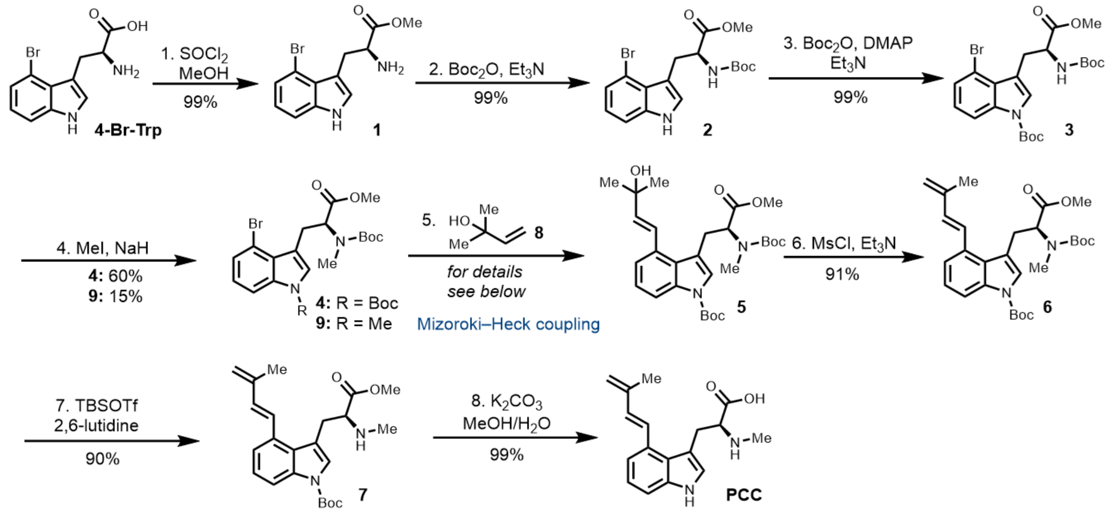

Reagents and conditions:

(1) $SOCl_2$ (3 equiv.), MeOH, 0 °C to 50 °C, overnight, 99%; (2) $(Boc)_2O$ (1.5 equiv.), Et_3N (2.0 equiv.), CH_2Cl_2, rt, 1 h, 99%; (3) $(Boc)_2O$ (1.5 equiv.), Et_3N (2.0 equiv.), DMAP (0.1 equiv.), CH_2Cl_2, rt, 2 h, 99%; (4) NaH (2.0 equiv.), MeI (2.0 equiv.), DMF, rt, 1-2h, **4:** 60%, **9:** 15%; (5) **8** (10 equiv.) $Pd(OAc)_2$ (0.1 equiv.), Ag_2CO_3 (0.6 equiv.), toluene, 90 °C, 71%; (6) Et_3N (6.0 equiv.), MsCl (3.0 equiv.), THF, 0 °C to rt, 91%; (7) TBSOTf (4.0 equiv.), 2,6-lutidine (5.0 equiv.), CH_2Cl_2, 0 °C, 90%; (8) K_2CO_3 (3.0 equiv.), $MeOH/H_2O$, 100 °C, 6 h, 99%.

Experimental:

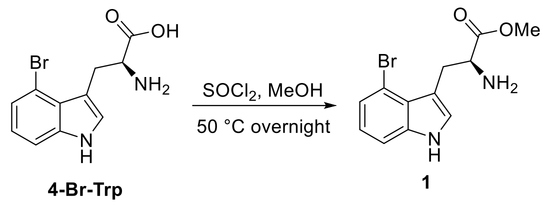

To a 50 mL flask equipped with a stir bar, 4-Br-Trp (0.25 mmol) was dissolved in methanol (10 mL) and cooled by ice-water. $SOCl_2$ (0.75 mmol, 3.0 equiv.) was added dropwise to the solution and then warmed to 50 °C. The reaction was stirred at this temperature overnight and then evaporated. Subsequently, the residue was suspended in sat. $NaHCO_3$ and extracted with CH_2Cl_2 (3×20 mL). The organic layer was dried over Na_2SO_4 and evaporated to afford the amino acid methyl ester hydrochloride **1** as a white solid at 99% yield. [1]

^{1}H NMR (400 MHz, Chloroform-*d*) δ 9.39 (s, 1H), 7.53 -7.20 (m, 2H), 7.03 - 6.93 (m, 2H), 4.05 (dd, *J* = 8.8, 5.2 Hz, 1H), 3.76 (s, 3H), 3.71 (dd, *J* = 14.4, 4.8 Hz, 1H), 3.05 (dd, *J* = 14.4, 9.2 Hz, 1H).

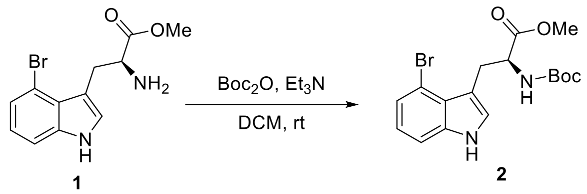

The amino acid methyl ester **1** (0.25 mmol) was dissolved in CH_2Cl_2 (20 mL), and Et_3N (0.5 mmol, 2.0 equiv.) was added, followed by $(Boc)_2O$ (0.75 mmol, 1.5 equiv.). The resulting solution was stirred at room temperature for 2 h and then washed with brine (20 mL). The organic layer was dried over Na_2SO_4 and evaporated. The residue was purified by petroleum ether elution to afford **2** as a white solid at 99% yield. [1]

^{1}H NMR (400 MHz, Chloroform-*d*) δ 8.33 (s, 1H), 7.34 (d, *J* = 8.4 Hz, 2H), 7.14 (s, 1H), 7.05 (t, *J* = 7.6 Hz, 1H), 5.23 - 5.07 (m, 1H), 4.79 - 4.69 (m, 1H), 3.76 (s, 3H), 3.73 - 3.63 (m, 1H), 3.44 (dd, *J* = 14.4, 8.4 Hz, 1H), 1.40 (s, 9H).

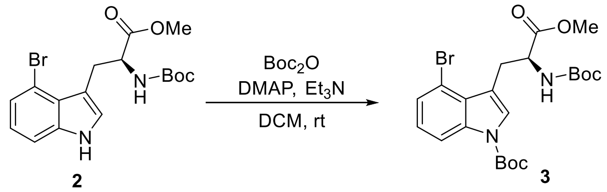

In a 50 mL flask, **2** (0.88 mmol) was dissolved in CH_2Cl_2 (10 mL), and Et_3N (1.32 mmol, 1.5 equiv.), DMAP (0.09 mmol, 0.1 equiv.) was added, and then followed by $(Boc)_2O$ (1.32 mmol, 1.5 equiv.). The reaction solution was stirred at room temperature for 2 hours and then washed with brine. The organic layer was dried over Na_2SO_4 and evaporated. The residue was purified with silica column chromatography and afforded **3** as a white solid at 99% yield. [1]

^{1}H NMR (400 MHz, DMSO-d_6) δ 8.13 (d, *J* = 8.4 Hz, 1H), 7.64 (s, 1H), 7.48 (d, *J* = 7.6 Hz, 1H), 7.35 (d, *J* = 8.4 Hz, 1H), 7.25 (t, *J* = 8.0 Hz, 1H), 4.51 - 4.41 (m, 1H), 3.68 (s, 3H), 3.62 - 3.53 (m, 1H), 3.07 - 2.96 (m, 1H), 1.64 (s, 9H), 1.30 (s, 8H), 1.09 (s, 1H).

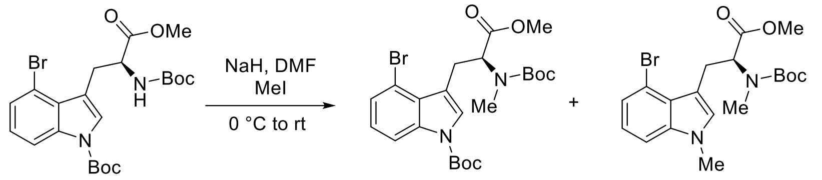

3 **4** **9**

To a solution of **3** (0.88 mmol) in DMF (6.0 mL) was added NaH (60% oil dispersion, 1.76 mmol, 2.0 equiv.) at 0 °C, and the mixture was stirred for 30 min. Then MeI (1.76 mmol, 2.0 equiv.) was added, and the reaction mixture was stirred at room temperature for 1-2 h. Saturated NH_4Cl, was added to the reaction followed by extraction with EtOAc. The combined organic phases were washed with brine and dried over Na_2SO_4. The residue was purified with silica column chromatography and afforded **4** as a foam solid in 60% yield and by-product **9** in 15% yield. [2]

Compound 4: **^{1}H NMR** (400 MHz, Chloroform-*d*) δ 8.28 - 8.12 (m, 1H), 7.51 - 7.35 (m, 2H), 7.21 - 7.11 (m, 1H), 5.13 - 4.96 (m, 1H), 3.90 - 3.70 (m, 4H), 3.17 - 3.05 (m, 1H), 2.76 (d, *J* = 17.6 Hz, 3H), 1.67 (s, 9H), 1.44 (s, 3H), 1.18 (s, 6H).

Compound 9: **^{1}H NMR** (400 MHz, Chloroform-*d*) δ 7.25 (d, *J* = 8.0 Hz, 1H), 7.20 (d, *J* = 8.0 Hz, 1H), 7.02 (dd, *J* = 8.0, 8.0 Hz, 1H), 6.94 (s, 0.3H, rotate isomer), 6.83 (s, 0.7H), 5.04 (dd, *J* = 8.0, 4.0 Hz, 1H), 3.87 (dd, *J* = 16.0, 4.0 Hz, 1H), 3.76 (s, 3H), 3.68 (s, 3H), 3.05 (dd, *J* = 15.0, 12.0 Hz, 1H), 2.76 (s, 2.2H), 2.72 (s, 0.8H, rotate isomer), 1.42 (s, 3H, rotate isomer), 1.08 (s, 6H).

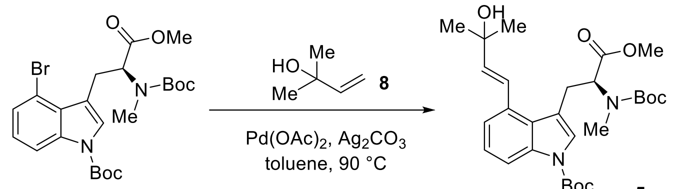

4 **5**

A suspension of **4** (198 mg, 0.307 mmol), 2-methyl-3-buten-2-ol **8** (264 mg, 3.07 mmol), $Pd(OAc)_2$ (7.0 mg, 0.031 mmol), and Ag_2CO_3 (50.7 mg, 0.184 mmol) in toluene (2.0 mL) was heated at 90 °C for 6 h and cooled to room temperature. The insoluble material was filtrated and washed with EtOAc. The filtrate was evaporated to dryness and purified with silica column chromatography to afford the desired **5** at 71% yield. [3]

^{1}H NMR (400 MHz, DMSO-d_6) δ 8.00 (d, *J* = 6.8 Hz, 1H), 7.40 (d, *J* = 12.8 Hz, 1H), 7.35 - 7.35 (m, 2H), 7.13 (d, *J* = 16.0 Hz, 1H), 6.28 (d, *J* = 15.6 Hz, 1H), 4.94 - 4.80 (m, 1H), 4.73 (s, 1H), 3.72 (d, *J* = 10.8 Hz, 3H), 3.44 (d, *J* = 15.2 Hz, 1H), 3.33 - 3.14 (m, 1H), 2.66 - 2.61 (m, 3H), 1.61 (s, 9H), 1.31 (d, *J* = 6.6 Hz, 9H), 0.95 (s, 6H).

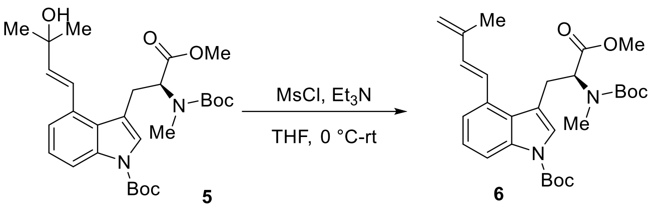

5 **6**

To a solution of the alcohol **5** (0.18 mmol) in anhydrous THF (1.0 ml) and Et_3N (1.1 mmol, 6.0 equiv.) under N_2 atmosphere was added MsCl (0.54 mmol, 3.0 equiv.) slowly at 0 °C. The solution was allowed to warm to room temperature for about 1.5 h and then refluxed for 30 min. The precipitate formed was filtered off using ethyl acetate affording a brown viscous liquid. Evaporation of the solvent and purification of the residue on silica gel column using EtOAc-hexane as eluent furnished the diene **6** at 91% yield. [4]

^{1}H NMR (400 MHz, Chloroform-*d*) δ 8.23 (d, *J* = 6.8 Hz, 1H), 7.45 - 7.34 (m, 2H), 7.33 - 7.25 (m, 1H), 7.20 (d, *J* = 16.0 Hz, 1H), 6.86 (d, *J* = 16.0 Hz, 1H), 5.23 - 5.08 (d, *J* = 15.2 Hz, 2H), 4.91 - 4.77 (m, 1H), 3.79 (s, 3H), 3.68 - 3.56 (m, 1H), 3.26 - 3.12 (m, 1H), 2.73 (d, *J* = 27.6 Hz, 3H), 2.01 (d, *J* = 5.6 Hz, 3H), 1.68 (s, 9H), 1.45 (s, 4H), 1.16 (s, 5H).

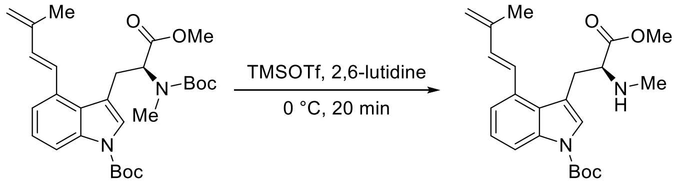

6 **7**

To a stirring solution of **6** (0.02 mmol) and 2,6-lutidine (0.10 mmol, 5.0 equiv.) in CH_2Cl_2 (0.2 mL) at 0 °C was added TMSOTf (0.08 mmol, 4.0 equiv.) dropwise. The reaction mixture was stirred at 0 °C for 20 min, and sat. NH_4Cl was added followed by extraction with EtOAc. The combined organic phases were washed with sat. $NaHCO_3$ and brine and dried over Na_2SO_4. Evaporation of the solvent and purification of the residue on silica gel column using EtOAc-hexane as eluent furnished the diene **7** at 90% yield. [3]

^{1}H NMR (400 MHz, Chloroform-*d*) δ 8.13 (d, *J* = 8.0 Hz, 1H), 7.45 (s, 1H), 7.39 (d, *J* = 7.6 Hz, 1H), 7.32 – 7.22 (m, 2H), 6.86 (d, *J* = 16.0 Hz, 1H), 5.15 (d, *J* = 15.6 Hz, 2H), 3.71 (s, 3H), 3.58 (t, *J* = 7.6 Hz, 1H), 3.28 (dd, *J* = 14.8, 6.4 Hz, 1H), 3.11 (dd, *J* = 14.8, 8.0 Hz, 1H), 2.36 (s, 3H), 2.08 (s, 3H), 1.67 (s, 9H).

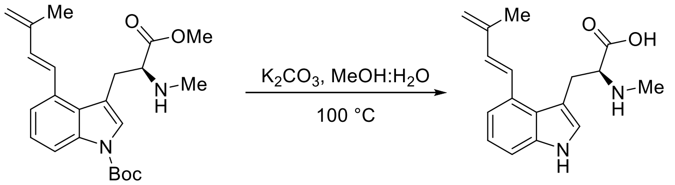

7 **PCC**

To a stirring suspension of **7** (22.5 mg, 0.056 mmol) and K_2CO_3 (23.4 mg, 0.169 mmol) in MeOH (1.2 mL) was added water (0.6 mL). The resultant reaction mixture was stirred at 100 °C for 6 h.

The solvent was removed under reduced pressure, and the residue was suspended in MeOH: DCM (4:1), filtered, and the filtrate was concentrated under vacuum to give **PCC** at 99% yield. [3]

^{1}H NMR (400 MHz, DMSO-d_6) δ 11.27 (s, 1H), 7.49 (d, *J* = 15.6 Hz, 1H), 7.30 - 7.15 (m, 3H), 7.01 (t, *J* = 7.6 Hz, 1H), 6.87 (d, *J* = 15.6 Hz, 1H), 5.09 (d, *J* = 40.4 Hz, 2H), 3.32 (d, *J* = 12.4 Hz, 1H), 3.04 (s, 1H), 2.96 (d, *J* = 12.8 Hz, 1H).

References:

1. Wu, H.; Yang, J.; Šečkutė, J.; and Devaraj, N. K. *Angew. Chem. Int. Ed.* **2014**, *53*, 5805-5809.
2. Ashworth, P.; Broadbelt, B.; Jankowski, P.; Kocienski, P.; Pimm, A.; Bell, R. *Synthesis* **1995**, *1995*, 199-206.
3. Xu, Z.; Hu, W.; Liu, Q.; Zhang, L.; and Jia, Y. *J. Org. Chem.* **2010**, *75*, 7626-7635.
4. Dethe, D. H.; Erande, R. D.; and Ranjan, A. *J. Am. Chem. Soc.* **2011**, *133*, 2864-2867.

^{1}H NMR spectra

Compound **1**

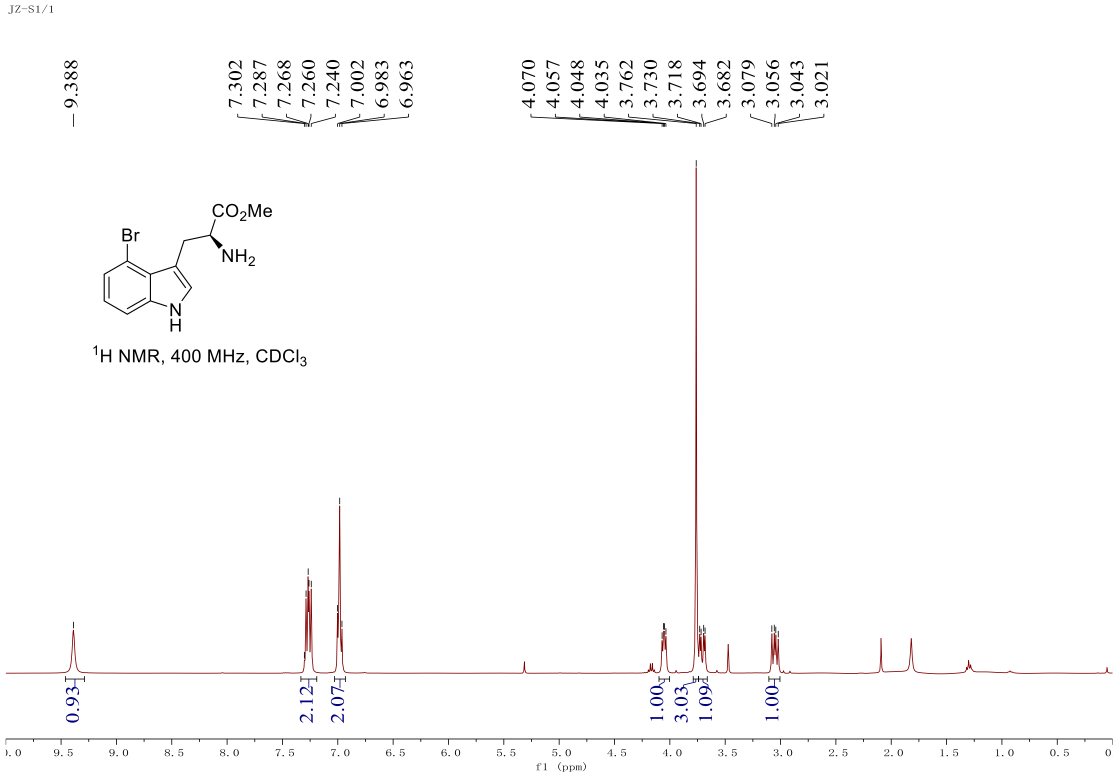

Compound **2**

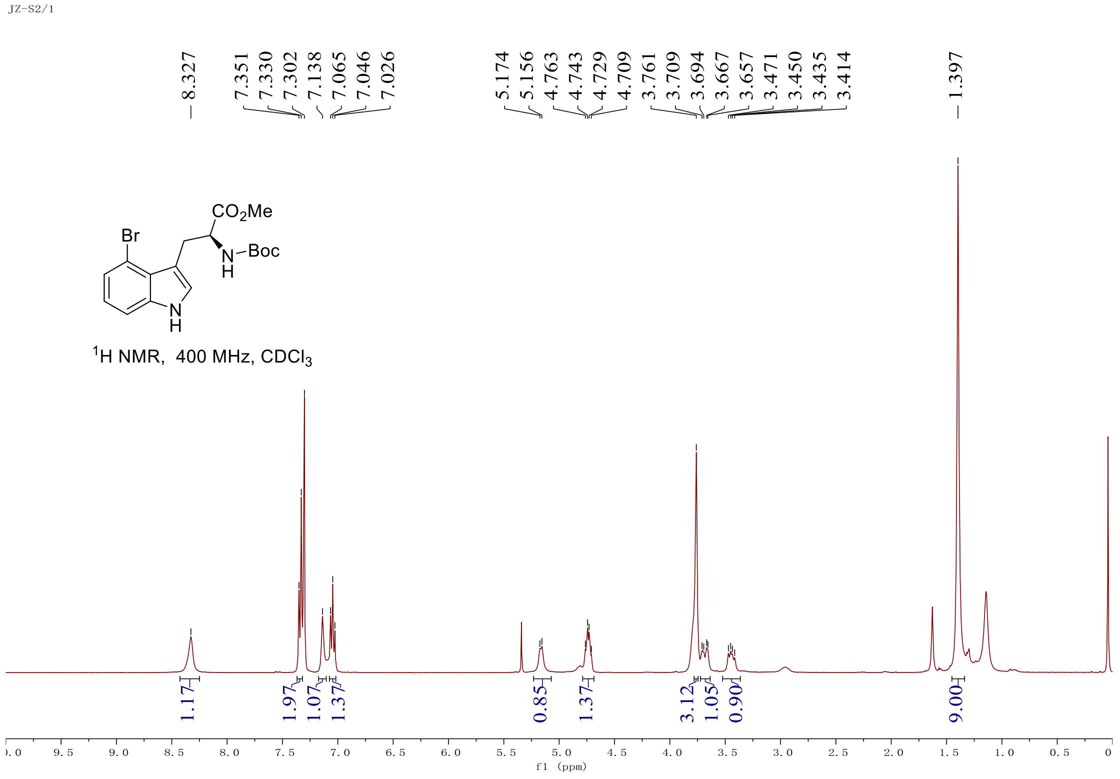

Compound **3**

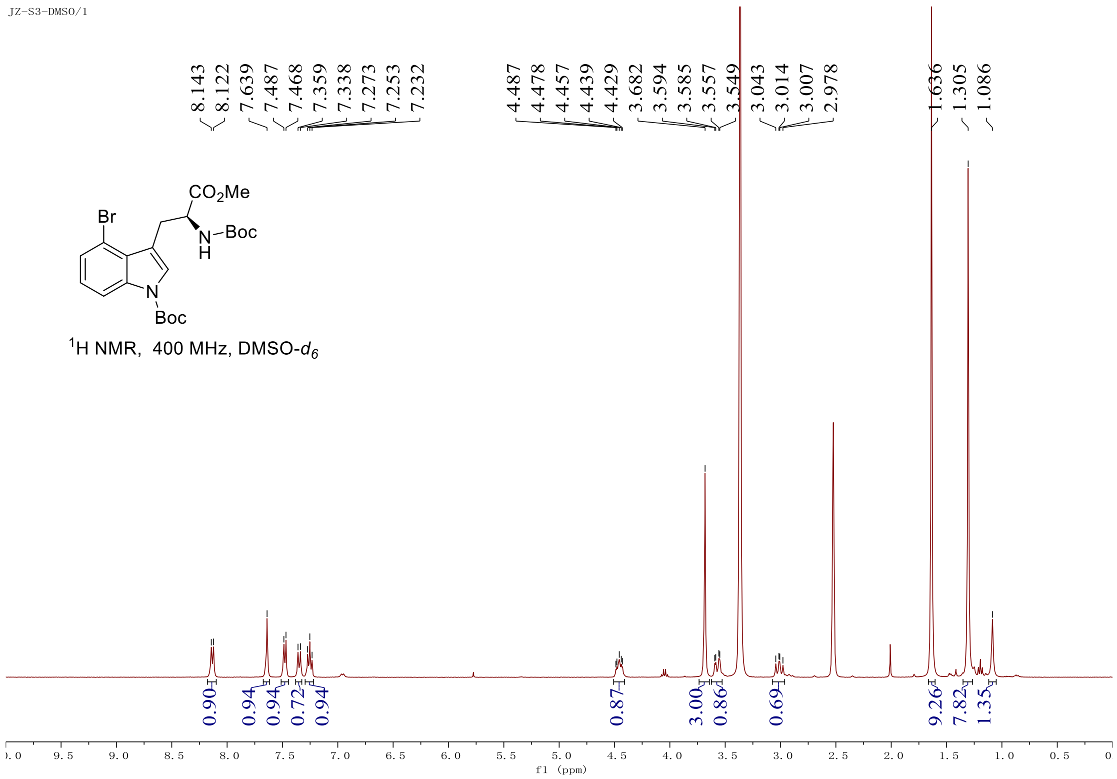

Compound **4**

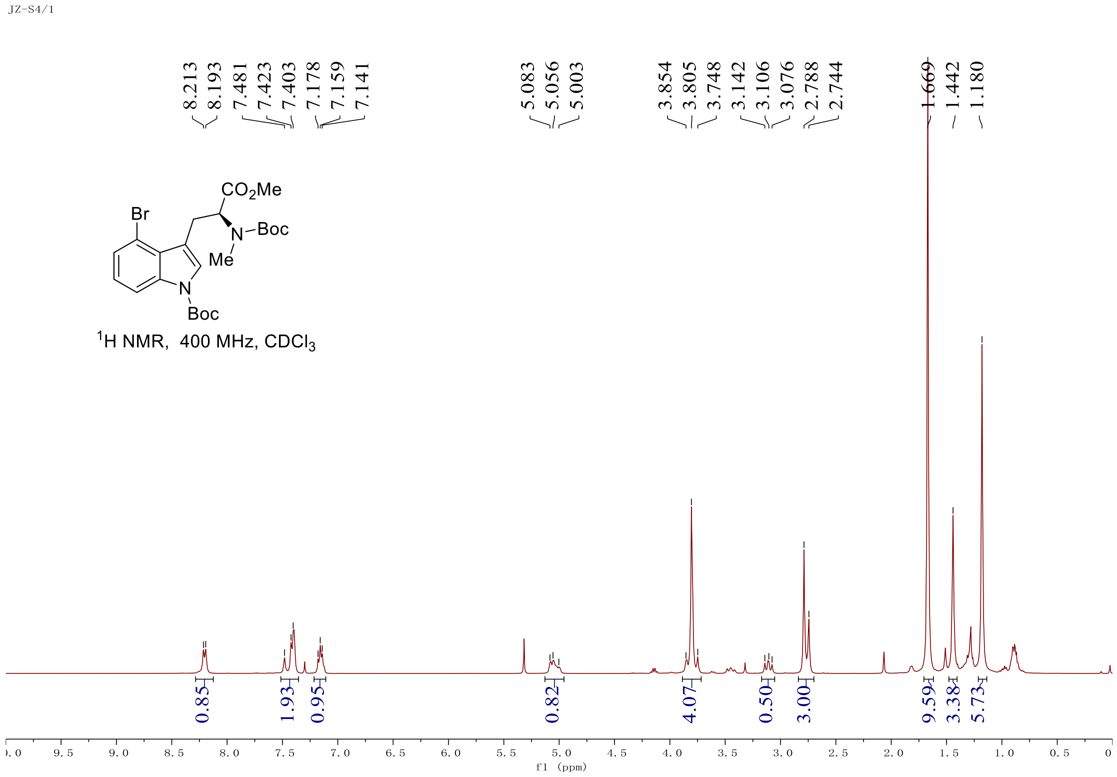

Compound **9**

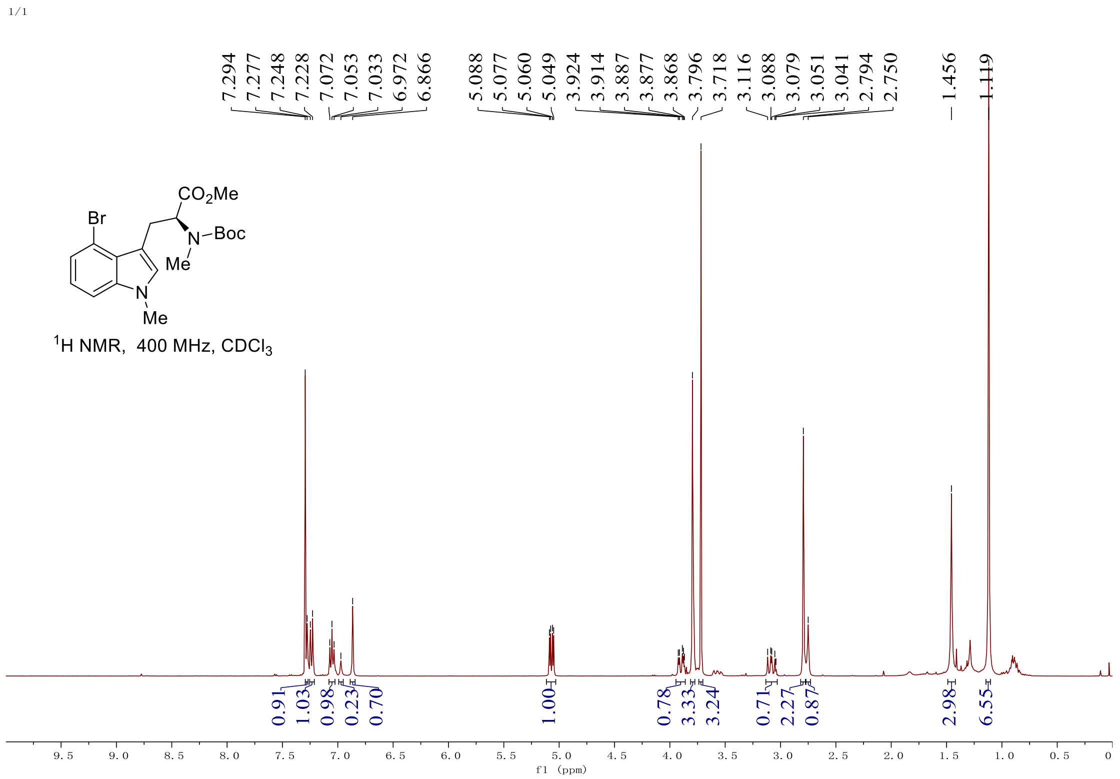

LCMS result for compound **9**

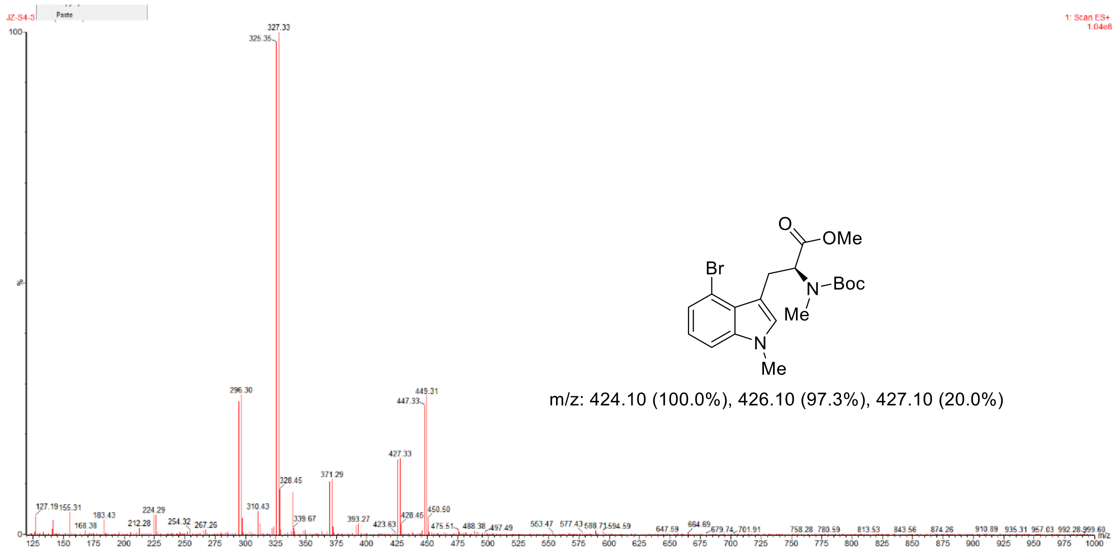

Compound **5**

JZ-S5-DMSO/1

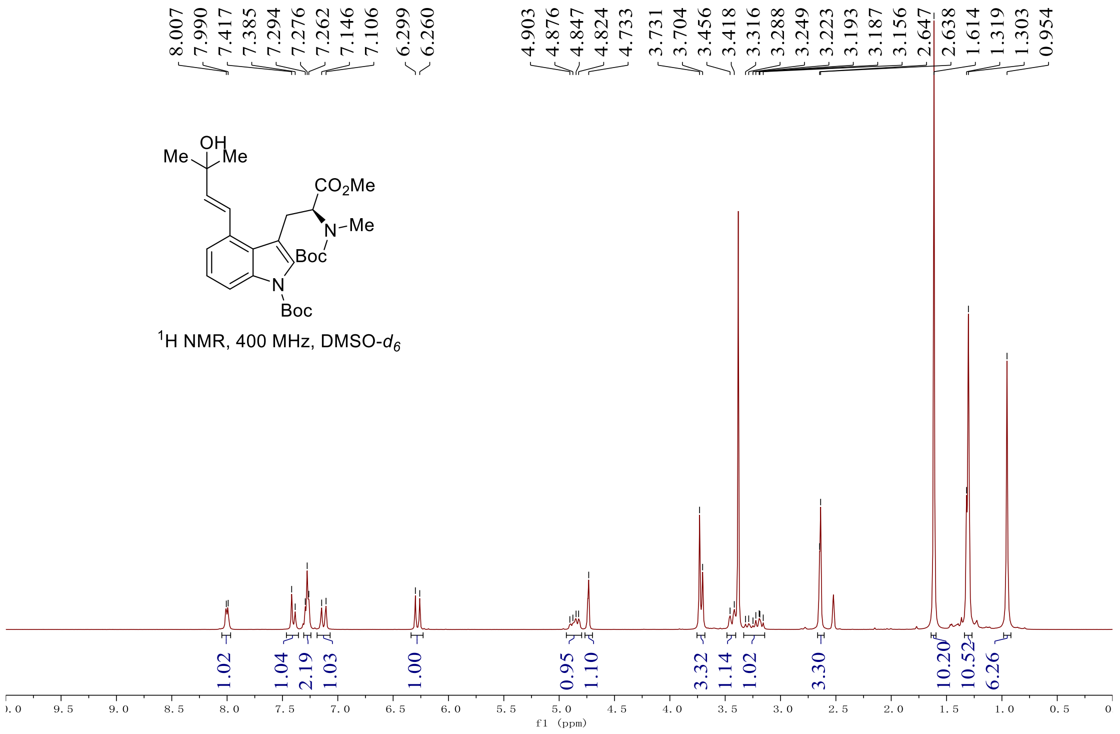

Compound **6**

JZ-S6/1

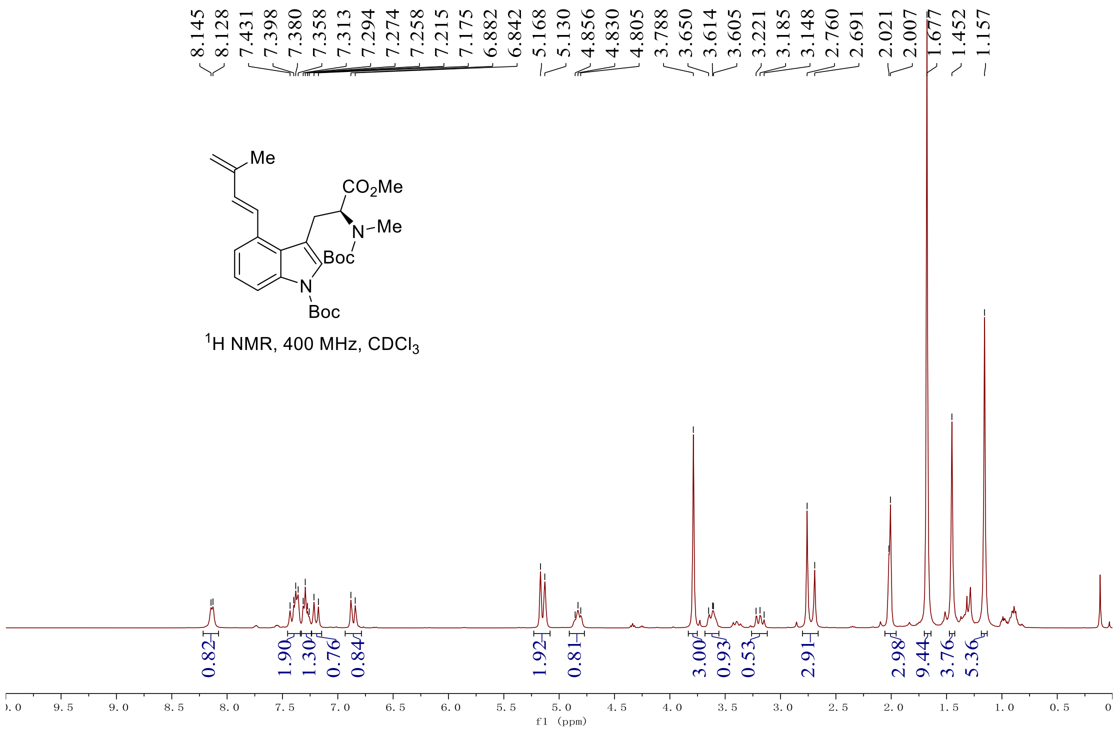

Compound **7**

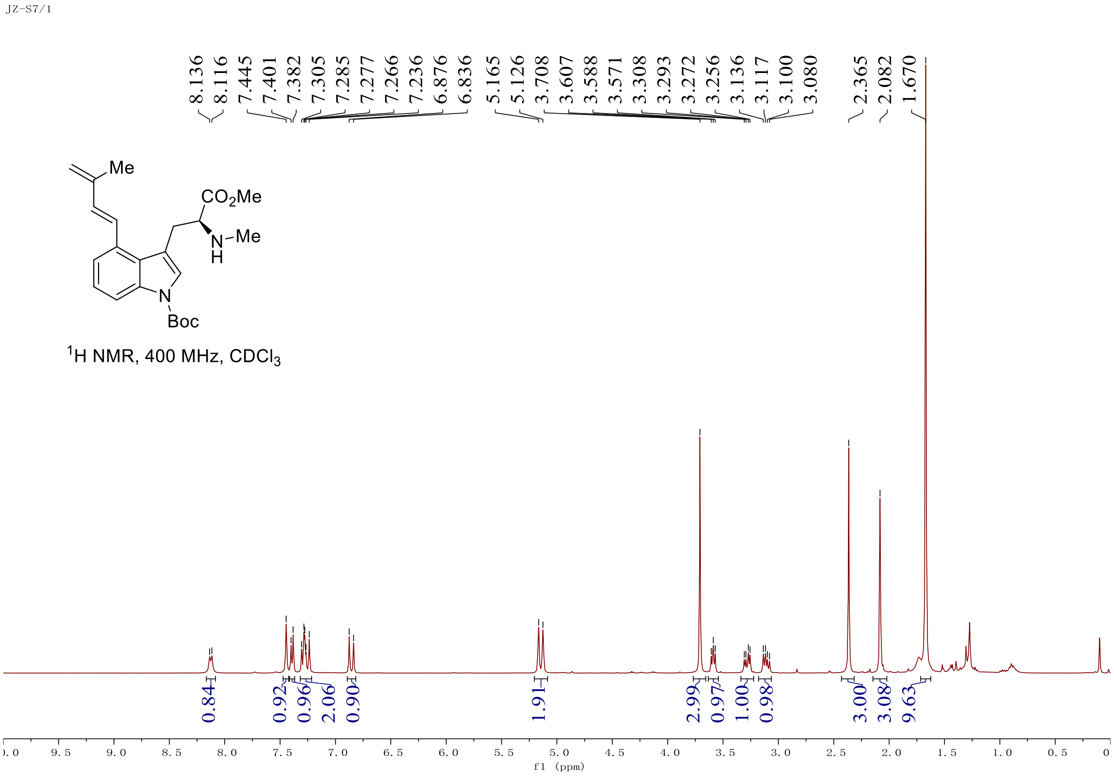

PCC

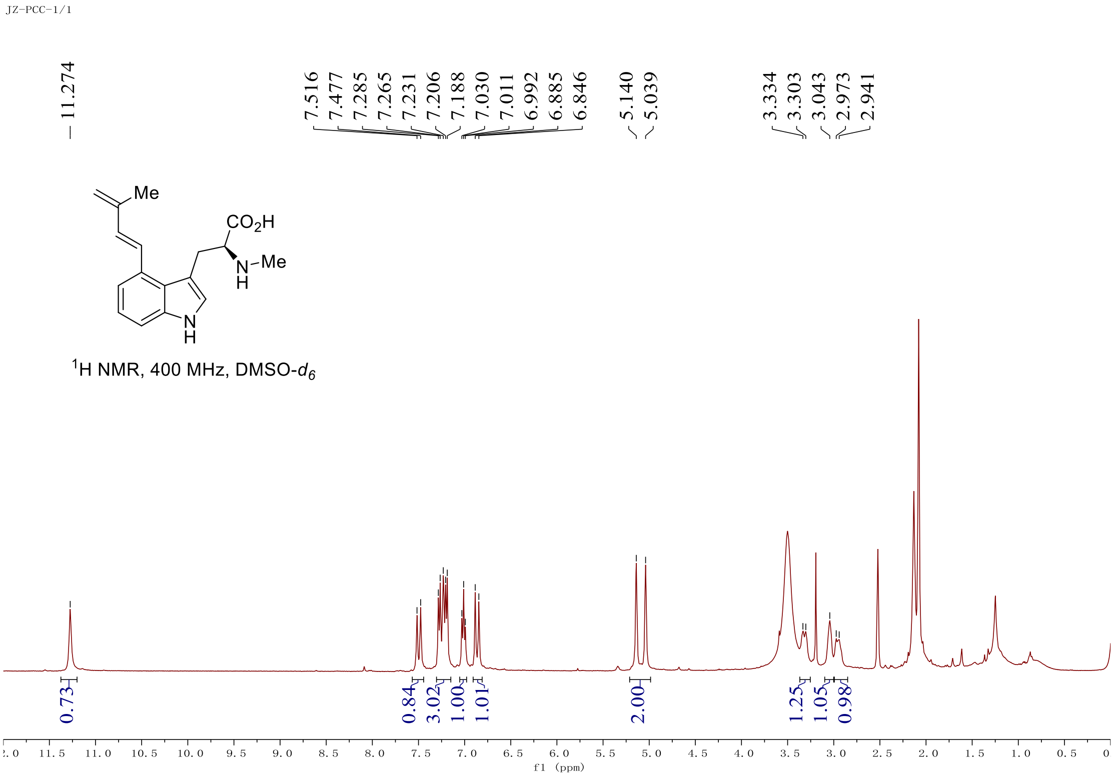

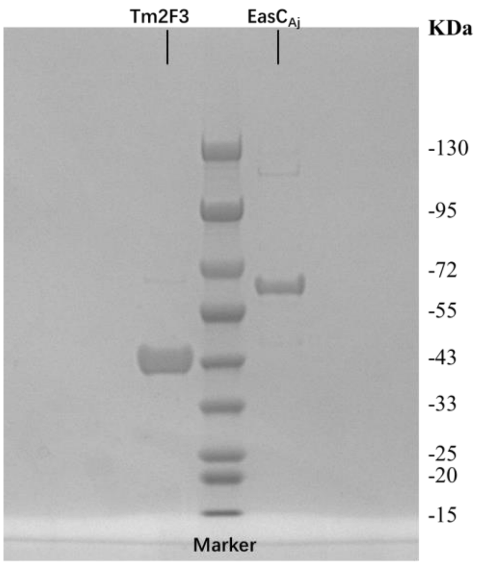

Figure S1 The SDS-PAGE result of purified Tm2F3 and EasC$_{aj}$.
